# Foodborne bacteria in milk and milk products along the water buffalo milk chain in Bangladesh

**DOI:** 10.1038/s41598-024-67705-3

**Published:** 2024-07-19

**Authors:** Shuvo Singha, Gerrit Koop, Md. Mizanur Rahman, Fabrizio Ceciliani, Md. Matiar Rahman Howlader, Sofia Boqvist, Paola Cremonesi, M. Nazmul Hoque, Ylva Persson, Cristina Lecchi

**Affiliations:** 1 Department of Veterinary Medicine and Animal Sciences, Università degli Studi di Milano, 26900 Lodi, Italy; 2 Department of Physiology, Veterinary, Animal and Biomedical Sciences, Sylhet Agricultural University, Sylhet, 3100 Bangladesh; 3 Department of Medicine and Surgery, Chattogram Veterinary and Animal Sciences University, Chattogram, 4225 Bangladesh; 4 Udder Health Bangladesh, Chattogram, 4225 Bangladesh; 5 Sustainable Ruminant Health, Department of Population Health Sciences, Faculty of Veterinary Medicine, Utrecht University, Utrecht, 3584 CL The Netherlands; 6 Department of Animal Biosciences, The Swedish University of Agricultural Sciences, 750 07 Uppsala, Sweden; 7 Institute of Agricultural Biology and Biotechnology, National Research Council, 26900 Lodi, Italy; 8 Department of Gynecology, Obstetrics and Reproductive Health, Bangabandhu Sheikh Mujibur Rahman Agricultural University, Gazipur, 1706 Bangladesh; 9 Swedish Veterinary Agency, 751 89 Uppsala, Sweden

**Keywords:** Pathogens, Epidemiology

## Abstract

Controlling foodborne pathogens in buffalo milk is crucial for ensuring food safety. This study estimated the prevalence of nine target genes representing seven critical foodborne bacteria in milk and milk products, and identified factors associated with their presence in buffalo milk chain nodes in Bangladesh. One hundred and forty-three milk samples from bulk tank milk (n = 34), middlemen (n = 37), milk collection centers (n = 37), and milk product shops (n = 35) were collected and analyzed using RT-PCR. *Escherichia (E.) coli*, represented through *yccT* genes, was the most prevalent throughout the milk chain (81–97%). Chi-squared tests were performed to identify the potential risk factors associated with the presence of foodborne bacteria encoded for different genes. At the middleman level, the prevalence of *E. coli* was associated with the Mymensingh, Noakhali, and Bhola districts (P = 0.01). The prevalence of *Listeria monocytogenes*, represented through *inlA* genes, and *Yersinia (Y.) enterocolitica*, represented through *yst* genes, were the highest at the farm level (65–79%). The prevalence of both bacteria in bulk milk was associated with the Noakhali and Bhola districts (P < 0.05). The prevalence of *Y. enterocolitica* in bulk milk was also associated with late autumn and spring (P = 0.01) and was higher in buffalo-cow mixed milk than in pure buffalo milk at the milk collection center level (P < 0.01). The gene *stx2* encoding for Shiga toxin-producing (STEC) *E. coli* was detected in 74% of the milk products. At the middleman level, the prevalence of STEC *E. coli* was associated with the use of cloths or tissues when drying milk containers (P = 0.01). *Salmonella enterica*, represented through the presence of *invA* gene, was most commonly detected (14%) at the milk collection center. The use of plastic milk containers was associated with a higher prevalence of *Staphylococcus aureus*, represented through *htrA* genes, at milk product shops (P < 0.05). These results suggest that raw milk consumers in Bangladesh are at risk if they purchase and consume unpasteurized milk.

## Introduction

Foodborne diseases affect about 600 million people annually, resulting in 0.4 million deaths yearly, including 0.1 million children under five^1^. Food-producing animals may act as a reservoir for many foodborne pathogens, and food products serve as vehicles for transmitting these pathogens to humans^2,3^. Human infections primarily result from ingesting foodstuffs either contaminated with pathogenic microorganisms or intoxicated by microbial toxins ^4^. Bacteria are responsible for two-thirds of human foodborne diseases, with a relatively heavy burden affecting low and middle-income countries (LMIC)^5–7^. Foodborne diseases also have economic consequences for healthcare systems, food producers, and distributors, and require specific attention from regulatory authorities^5,8^. Despite the increased global awareness of foodborne infections as a threat to public health and socio-economic development, food safety requires further attention, specifically focusing on reducing pathogen spillover in LMIC.

Milk and milk products are nutritionally rich and are considered to be an important component of many healthy diets. However, if contaminated, they can be a source of pathogenic microorganisms^9^. Milk is a suitable growth medium for many microorganisms due to its high nutritional value stemming from its proteins, sugars, and lipid content^10,11^. Buffalo milk is rich in fat and protein. It is, therefore, used for ghee and cheese preparation and is well accepted by consumers^12–14^. Buffalo milk in Bangladesh has been reported to harbour many microbes, including lactic acid bacteria, spoilage, and pathogenic organisms^15–17^. Previous studies in Iran, Sweden, and Brazil have evidenced the presence of foodborne bacteria in milk from cows, sheep, and goats^18–20^. Although buffalo milk accounts for 35% of the total milk production of Asian countries^21^, there are limited reports^22,23^ estimating the prevalence of foodborne pathogens from buffalo milk in LMIC.

Bangladesh's water buffalo rearing system primarily includes free-range systems on coastal or semi-coastal islands, and a semi-intensive or intensive buffalo rearing system in the inlands. Buffalo milk trading also includes different levels of handlers, such as middlemen, milk collection centers, and milk product shops^24,25^. The buffalo milk chain in Bangladesh is a supply chain consisting of activities and processes including the production, processing, trading, and consumption of milk and milk products^26,27^. Farms are typically situated far from the milk processing centers and hygienic practices in milk handling are minimal. Consequently, when local transportation is prolonged and labor-intensive measures are required, the absence of milk cooling facilities can compromise milk safety due to potential contamination by foodborne pathogens^28^. Previous studies have identified zoonotic microorganisms, including *Staphylococcus* (*S.*) *aureus*, *Escherichia* (*E.*) *coli*, and enteropathogenic *E. coli* O157:H7 from bovine bulk milk and cheese samples^29–31^. *Staphylococcus* spp. are the most prevalent mastitis-causing pathogens isolated from water buffalo milk samples^32,33^. The enteropathogenic properties of *E. coli* O157:H7 are associated with enterohemorrhagic diseases in humans. Enteropathogenicity from *E. coli* is exhibited as Shiga toxin-producing *E. coli* (STEC), which corresponds mainly to the genes *stx1* and *stx2*^34^. Shiga toxin-producing *E. coli* has been identified in children, likely through household livestock fecal contamination through foodborne routes^35^. *Campylobacter* (*C.*) *jejuni*, *Listeria* (*L.*) *monocytogenes*, *Salmonella* spp., and *Yersinia* (*Y.*) *enterocolitica* were also previously identified in cow bulk milk and milk products^30,36–38^. Thus, water buffalo milk and milk products may be a source for transmitting foodborne pathogens to humans. Guaranteeing an adequate hygienic status at the farms and during milk handling along the stages of production is required to achieve better quality and safety in milk and milk-derived products. A considerable obstacle to adequately addressing food safety concerns is the lack of data. For example, data on the prevalence of foodborne pathogen contamination in buffalo milk and milk products, enabling policymakers to set public health priorities and allocate resources, is needed. Knowing the risk factors associated with the prevalence of foodborne pathogens may help identify effective control measures to reduce the introduction of such pathogens into the food chain and ensure the public health safety of buffalo milk consumers in Bangladesh. To meet this need, the present study aims to estimate the prevalence of nine target genes belonging to seven critical foodborne bacteria, *S. aureus*, *E. coli*, Shiga toxin-producing *E. coli*, *C. jejuni*, *L. monocytogenes*, *Salmonella* (*S.*) *enterica*, and *Y. enterocolitica,* using molecular methods and to identify the factors associated with these bacteria in milk and milk products along the buffalo milk chain in Bangladesh.

## Results

### Prevalence of foodborne bacteria along the buffalo milk chain

**Figure 1:**
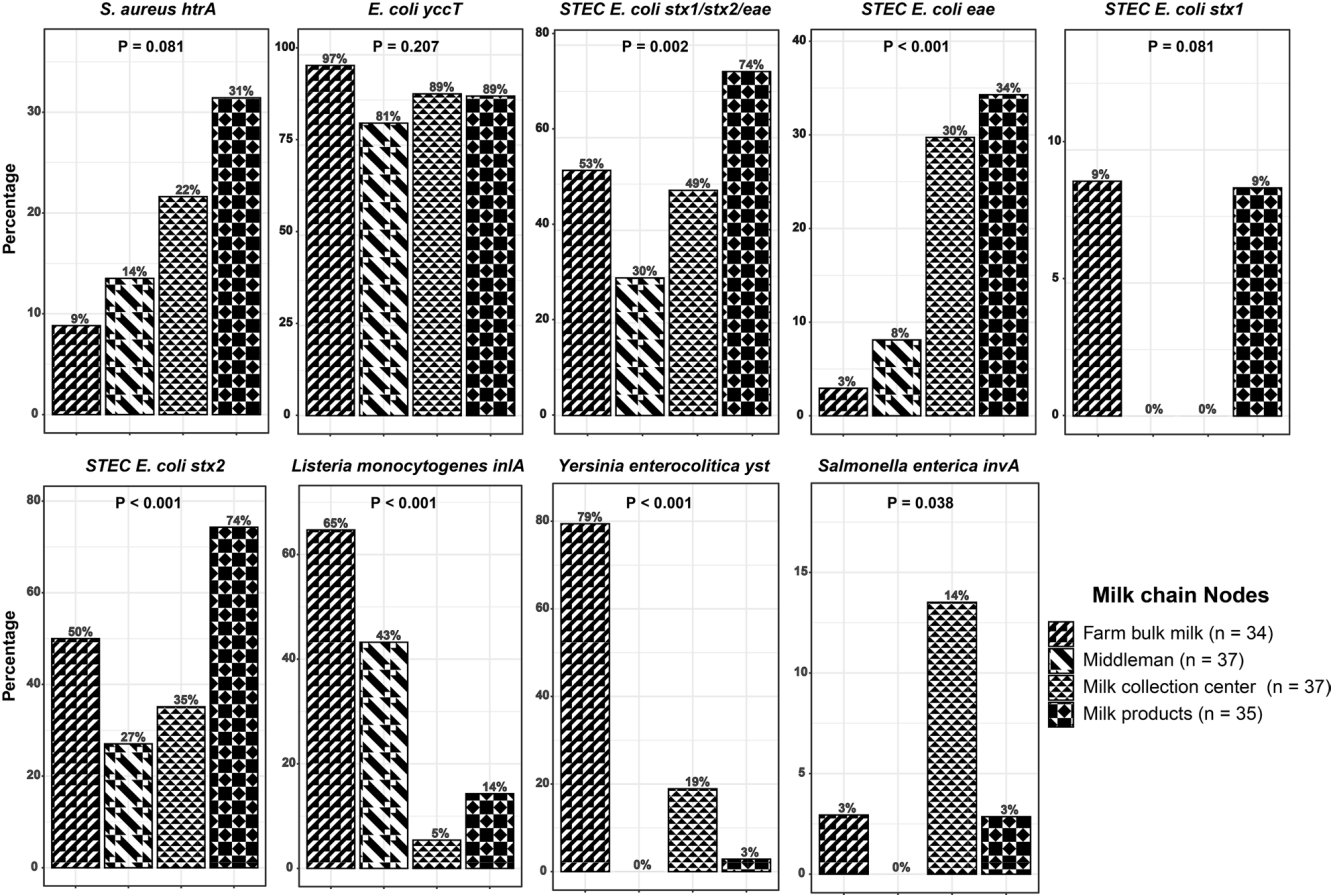
The prevalence of target genes for most of the foodborne bacteria was very high in the buffalo milk chain in Bangladesh. Figures show the prevalence of the RT-PCR identified target genes for foodborne bacteria from the milk and milk product samples (N = 143) collected from six districts in Bangladesh with a high density of buffalo. The P value was obtained from a chi-squared test by comparing each pathogen prevalence from the samples from four buffalo milk chain nodes (farm bulk milk, middleman, milk collection center, and milk product).

The overall prevalence of RT-PCR identified foodborne bacteria in milk and milk products was very high. Figure 1 shows that *E. coli,* represented through the presence of *yccT* genes, was the most prevalent (81–97%) pathogen in the buffalo milk chain and had an exceptionally high prevalence in farm bulk milk. The presence of STEC *E. coli* virulence-specific genes significantly differed (P < 0.001) along the milk chain and was higher in milk products than at the farm level. The prevalence of *S. aureus*, represented through *htrA* genes, increased along the milk chain (P = 0.08), but *L. monocytogenes,* represented through *inlA* genes, (P < 0.001) and *Y. enterocolitica* represented through *yst* genes, (P < 0.001) decreased along the milk chain and were extremely high in farm bulk milk. The prevalence of *S. enterica,* represented through *invA* genes, was low at the farm and middleman levels (0–3%) but higher at the milk collection center level (14%). *C. jejuni,* represented through *cadF* genes, was absent in all samples.

### Factors associated with the prevalence of foodborne bacteria in the buffalo milk chain

Several factors were associated with the higher prevalence of foodborne bacteria at buffalo milk chain nodes. Importantly, bulk milk from the buffalo farms in the Noakhali and Bhola districts had a higher prevalence of genes for *L. monocytogenes* and *Y. enterocolitica* than in the other districts (P < 0.05). The prevalence of *yst* genes for *Y. enterocolitica* was higher in late autumn and spring compared to winter (P = 0.01). One variable, “Keep bulk milk containers open” had a single missing value (Table 1). At the middleman level, three variables, “Type of container”, “Type of trader”, and “How the container is dried,” had missing values ranging between 3 and 16%. The prevalence of *yccT* genes for *E. coli* was higher in the Mymensingh, Noakhali, and Bhola districts than in Rajshahi and Jamalpur. The milk containers that were dried using cloths or tissues had a higher prevalence of genes for STEC *E. coli* than for sun-dried containers (Table 2)*.* At the milk collection center level, one variable, “Nature of milk,” had a single missing value. The prevalence of *yst* genes for *Y. enterocolitica* was higher in buffalo-cow mixed milk than in homogeneous buffalo milk. In the milk products, plastic milk containers had a higher prevalence of *htrA* genes for *S. aureus* than earthenware and glass-made containers (Table 3).

**Table 1:** The univariable association between variables associated with bulk milk and the prevalence of foodborne bacteria (P < 0.10) in 34 bulk milk samples collected from six districts with a high density of buffalo.

Variable	Category	N	*E. coli*		*STEC*^a^		*L. monocytogenes*		*Y. enterocolitica*	
			Positive (%)	*P*	Positive (%)	*P*	Positive (%)	*P*	Positive (%)	*P*
Season	Late autumn	11							11 (100.0)	0.01
	Spring	19							15 (78.9)	
	Winter	4							1 (25.0)	
District	Noakhali	11			6 (54.5)	< 0.01	9 (81.8)	0.08	9 (81.8)	0.02
	Bhola	5			5 (100.0)		4 (80.0)		5 (100.0)	
	Moulvibazar	4			3 (75.0)		2 (50.0)		1 (25.0)	
	Mymensingh	4			3 (75.0)		0 (0.0)		4 (100.0)	
	Jamalpur	6			0 (0.0)		4 (66.7)		6 (100.0)	
	Rajshahi	4			1 (0.25)		3 (75.0)		2 (50.0)	
Keep bulk milk containers open	Yes	30			18 (60.0)	0.08				
	No	3			0 (0.0)					
Previous history of clinical mastitis in the last 12 months	Yes	31	31 (100.0)	0.09						
	No	3	2 (66.7)							

^a^The prevalence of STEC was calculated based on whether at least 1 of the toxin genes (*stx1* or *stx2* or *eae*) was present.

**Table 2:** The univariable association between variables associated with the middleman and the prevalence of foodborne bacteria (P < 0.10) in 37 bulk milk samples collected from six districts with a high density of buffalo.

Variable	Category	N	*S. aureus*		*E. coli*		*STEC E. coli*	
			Positive (%)	*P*	Positive (%)	*P*	Positive (%)	*P*
District	Noakhali	20			19 (95.0)	0.01		
	Bhola	5			4 (80.0)			
	Moulvibazar	4			3 (75.0)			
	Mymensingh	3			3 (100.0)			
	Jamalpur	4			1 (25.0)			
	Rajshahi	1			0 (0.0)			
Season	Late autumn	7			4 (57.1)	0.06		
	Spring	6			4 (66.7)			
	Summer	20			19 (95.0)			
	Winter	4			3 (75.0)			
Type of container	Aluminum	13	4 (30.8)	0.05				
	Plastic	23	1 (4.3)					
Type of trader	Farmer or middleman	18			12 (66.7)	0.09		
	Wholesalers	16			15 (93.8)			
How the container is dried	Sundry	15					1 (6.7)	0.01
	Cloth or tissue	16					9 (56.3)

**Table 3:** The univariable association between variables associated with the milk collection center and milk product shop and the prevalence of foodborne bacteria (P < 0.10) in 72 samples collected from six districts with a high density of buffalo.

Milk chain node	Variable	Category	N	*S. aureus*		*Y. enterocolitica*	
				Positive (%)	*P*	Positive (%)	*P*
Milk collection center (n = 37)	Nature of milk	Buffalo milk	25			1 (4.0)	< 0.01
		Buffalo-cow mixed milk	11			6 (54.5)	
Milk product shop (n = 35)	Type of container	Earthen	10	2 (20.0)	0.04		
		Plastic	18	9 (50.0)			
		Glass	7	0 (0.0)			

## Discussion

Our study aimed to estimate the prevalence of and identify the risk factors associated with the presence of RT-PCR identified foodborne bacteria, namely *S. aureus*, *E. coli*, STEC *E. coli*, *L. monocytogenes*, *Y. enterocolitica*, *S. enterica*, and *C. jejuni*, in the water buffalo milk chain in Bangladesh. The prevalence of *yccT* genes for *E. coli* was high in farm bulk milk and decreased very little over the buffalo milk chains in our study. This is consistent with our previous study, which demonstrated the progressive increase of overall Enterobacteriaceae count in the water buffalo milk chain in Bangladesh^25^. *E. coli* is often used as an indicator of fecal contamination and is often regarded as commensal rather than pathogenic. However, high contamination levels of pathogenic *E. coli* can also influence pathogen colonization, leading to an increase in disease severity^39,40^, and may disseminate antimicrobial resistance genes^41,42^; thus, it should be considered a health hazard when consuming raw milk or milk products. The prevalence of *E. coli* in composite buffalo milk was shown to vary between 33 to 67% in earlier studies in Iraq^43^, Egypt^44^, and Indonesia^45^, which is comparatively lower than in our study. These studies used bacterial culture to represent the proportion of live bacteria, and the prevalence may be relatively lower than when detected using RT-PCR without culture. Sample type may be another source of variation for the relatively lower prevalence of *E. coli* in these studies, as bulk milk could be more likely to be contaminated by external contaminants from the environment than composite milk; however, no information was found on the minorly decreasing prevalence of *E. coli* in the buffalo milk chain, e.g., at the middleman or milk collection centre levels. This can partly be explained by the fact that previous studies in Italy showed that the freezing of raw milk and the heat treatment applied during stretching for Mozzarella cheese preparation may reduce *E. coli* contamination in milk products^46,47^. We, therefore, suggest that enforcing rules to maintain the cold chain could help reduce *E. coli* contamination in the buffalo milk chain.

In this study, the prevalence of STEC *E. coli* virulence genes *eae*, *stx1*, and *stx2* increased along the buffalo milk chain; the prevalence of *stx2* was especially high in farms (50%) and milk products (74%). However, the prevalence of STEC *E. coli* was reasonably low in studies of water buffalo quarter milk in Turkey (1.4%) and Italy (0.6%)^48,49^. No previous study has evidenced the prevalence of STEC *E. coli* virulence genes in bulk milk in water buffalo. A recent survey in Bangladesh demonstrated a higher prevalence of *stx1*, *stx2,* and *eae* (40–57%) in cattle, poultry, and diarrheal human patients^50^. The present study found that the prevalence of STEC *E. coli* was higher in Noakhali and Bhola districts, where free-range buffalo rearing is more common. In buffalo farms in a free-ranging system, buffalo, cattle, sheep, and sometimes chickens and ducks are often reared in the same farm area. Moreover, buffalo farm workers mostly live close to the farm boundary. Therefore, there may be a risk of the spillover of foodborne bacteria carrying these virulence genes when humans have close interactions with livestock species. Plastic milk containers and milk handling at the middleman level were associated with a higher prevalence of *E. coli* O157:H7 in dairy cows^51^, confirming our findings. Plastic milk containers are more challenging to clean than glass or stainless-steel ones, and spoilt milk from the day before can easily be contaminated with pathogenic microorganisms. Using cloths or tissues to dry containers was more often related to a higher prevalence of STEC *E. coli* than sun-drying. The cloths used to clean and wipe the containers were unclean and frequently used without washing the containers in between. Occasionally, the staff used the cloths for their own personal use, meaning that they could contaminate milk during shipment if overused.

The prevalence of *htrA* genes of *S. aureus* in our study ranged between 9 and 31%, increasing along the buffalo milk chain. A recent study in Bangladesh has also reported an increasing level of *S. aureus* along the water buffalo milk chain^15,25^. In dairy cows, previous studies have shown that the prevalence of *S. aureus* was higher in cheese (33–40%) than in bulk milk (13–25%)^52,53^. The presence of *S. aureus* in bulk milk might be due to clinical or subclinical mastitis or unhygienic handling and milk processing^54^. Milk container material and drying methods were found to be risk factors for the presence of *htrA* genes of *S. aureus* at the middleman and milk product shop levels. The increasing *S. aureus* contamination levels at the middleman and milk product shop levels in the current study may be the outcome of the poor hygiene conditions practiced by middlemen and at collection centers during the handling of milk, as well as an insufficient cold chain that supports the exponential growth of previously introduced microorganisms at the milk producer level.

The prevalence of *inlA* genes of *L. monocytogenes* and *yst* genes of *Y. enterocolitica* was high in the buffalo farms but lower later in the milk chain. The prevalence of *inlA* genes for *L. monocytogenes* in this study (65%) is much higher than in water buffalo bulk milk in Pakistan, Egypt, and Brazil (0–8%)^22,55,56^. There were no reports on prevalence along the milk chain for these countries. However, this finding is consistent with the previous studies in dairy cows, which demonstrated that the prevalence of *L. monocytogenes* was higher in raw bulk milk (19–40%) than in traditional milk products, such as, yogurt, butter, and cheese (1–8%)^57,58^. Unlike many bacteria, *Listeria* may survive with exponential growth rates in low temperatures, ranging from 0 to 5 °C, but will start decreasing at 13 °C^59^. Therefore, the absence of a cold chain seems beneficial for reducing the prevalence of *L. monocytogenes* later in the milk chain in water buffalo in Bangladesh. *Listeria* may also survive in the natural environment, can be widely distributed in soil, and can contaminate roughage^60,61^. Water buffalo in Bangladesh primarily rely on grazing and often consume green roughage, which may be contaminated by soil. Thus, contamination might potentially occur in the bulk tank if the container is left open, contributing to the elevated prevalence of *L. monocytogenes* in the farm's bulk milk. In previous studies, the prevalence of *Y. enterocolitica* was also higher in the bulk milk from water buffalo (25%) than in milk products, such as commercial or traditional cheese and yogurt (0–12%)^62,63^, which is consistent with our study. We found that the prevalence of *yst* genes in *Y. enterocolitica* in bulk milk was higher in late autumn and spring. This may be linked to contamination during manual milk handling on farms in combination with favourable environmental conditions for the pathogen, given that the ambient temperature during late autumn and spring remains above 20 °C. An earlier study reported that *Y. enterocolitica* did not grow at a low pH (< 4.5) or in temperatures ranging from 5 to 19 °C^64^. Processing traditional buffalo milk products likely increases the acidity of the milk products, which may reduce the contamination level of *Y. enterocolitica* in buffalo milk products^62,63^.

*S. enterica,* represented through *invA* genes, was incredibly low (0–3%) at the farm and middleman level, and *C. jejuni,* represented through *cadF* genes, was absent in all the samples throughout the buffalo milk chain. It may be suggested that raw milk and milk production from the water buffalo milk chain is safe from contamination by *C. jejuni* in Bangladesh. These findings are consistent with a previous study of Bangladesh's dairy cow milk chain, which showed no evidence of *Salmonella* spp. or *C. jejuni*^65^. However, another recent study conducted in Ethiopia by Geletu et al.^66^ revealed that the prevalence of *Salmonella* spp. in bulk milk and milk collection centers ranged from 10 to 20% for dairy cows. This prevalence was higher than Brazil and India's 0% to 4% in buffalo milk^22,23^ and was comparable with our study. The prevalence of *C. jejuni* was mainly reported in bulk milk from dairy cows, ranging from 3 to 20% in different countries, including Italy, Tanzania, and Egypt^67–69^. However, there are limited studies for water buffalo, although Serraino et al.^69^ reported the absence of *C. jejuni* in water buffalo farms in Italy. No significant risk factors were identified for this study's higher prevalence of *invA* genes of *S. enterica*. However, a higher prevalence of *Y. enterocolitica* at the milk collection center level was more associated with cow-buffalo mixed milk than with pure buffalo milk. This finding is also consistent with a previous study^25^, indicating that combining milk from different sources may increase bacterial contamination in water buffalo milk in Bangladesh. Researchers have attempted to explore the implications of contamination for food safety. A study in South Korea describes an awareness survey and demonstrates that bacterial contamination and somatic cell counts were lower and milk solids, such as protein and fat content, were significantly higher in HACCP-certified farms than in non-certified farms^70^. Therefore, attention payed to the HACCP certification of farmers could help identify barriers to milk quality and contribute to a more sustainable and hygienic milk supply chain.

Our study found that the district of origin could be a risk factor, with the bulk milk samples collected from the Bhola district having a high prevalence of STEC, *Y. enterocolitica*, and *L. monocytogenes* (80–100%). This may be because free-range rearing relies primarily on grazing, which contaminates soil, and the farms in this district’s inferior transport facilities and remote locations require longer transportation time. Furthermore, milk handling hygiene was often poor. However, only a univariable analysis could be performed in this study because of the small number of samples analyzed for each sample type. Therefore, a further large-scale study is necessary to assess a more comprehensive number of variables to identify the most effective management practices for reducing foodborne pathogen contamination in Bangladesh's water buffalo milk chain.

This study estimated the prevalence of foodborne bacteria by detecting the selected genes which indicate the presence of various bacteria. However, confirmation by bacteriological culture is required to confirm the presence of viable bacteria in the samples. This is a limitation of our work, and studies using bacteriological culture are needed to overcome this limitation. Still, we feel that the variation in the prevalence of bacteria based on the detection of DNA through PCR is informative of which nodes in the dairy chain are most crucial. In addition, a negative PCR is likely to reflect the absence of viable bacteria, as the sensitivity of PCR is generally higher than for bacteriological culture^71,72^. The use of direct DNA extraction followed by PCR allows for a more rapid identification of targeted bacteria than bacteriological culture and is thus valuable for the almost real-time monitoring of the presence of bacteria in samples, but culture data is needed to confirm the actual human health hazards.

## Conclusions

Our findings give an indication that several potential human pathogenic bacteria, including STEC and *S. aureus*, are circulating throughout the buffalo milk chain in Bangladesh. This creates a major risk to public health and necessitates the establishment of suitable interventions in future studies. Targeted genes for foodborne bacteria are present at higher levels, starting at the farm level and increasing in the milk collection centers in the buffalo milk chain. Therefore, pathogenic bacteria in milk can be reduced by employing hygienic milking practices, replacing plastic milk containers with glass or stainless steel, and halting the use of dirty cloths when cleaning containers. Temperature-controlled milk containers could be introduced to reduce bacterial multiplication during transportation by middlemen. A small-scale pasteurization and chilling plant could be established at the milk collector level; this would effectively destroy pathogenic organisms that survive at low temperatures, such as *L. monocytogenes* and *Y. enterocolitica*. Finally, regular monitoring is required for the presence of foodborne bacteria. Pasteurization is recommended for raw milk to ensure the milk products are of better quality and to ensure the safety of consumers.

## Materials and methods

### Study design

This cross-sectional study was conducted between February 2020 and April 2021 at four buffalo milk chain nodes (farm, middleman, milk collection center, and milk product shop) in six districts in Bangladesh (Noakhali, Bhola, Moulvibazar, Mymensingh, Jamalpur, and Rajshahi). The list of farmers was created with the help of the Upazilla Veterinary Hospital (UVH) and a non-governmental organization named the Palli Karma-Sahayak Foundation working with buffalo farmers in Bangladesh. The study was approved by the Sylhet Agricultural University Research System (SAU/Ethical Committee/AUP/21/06) at the Sylhet Agricultural University, Bangladesh, and all methods were performed following the relevant guidelines and regulations. The buffalo farmers gave written informed consent, and middlemen, the managers of milk collection centers, and milk product shop owners gave oral consent before participating in this study. A list was created by collecting data on the numbers of buffalo farmers and local buffalo milk product shops at each study location, described in two previous studies in this project^25,73^. From there, the farms, middlemen, milk collection centers, and milk product shops were randomly recruited for this study based on the sample size estimation. The sample size was calculated at a 50% prevalence with a 95% confidence and an absolute margin of error of 0.10^74^. This required 35 samples, including 10 additional samples for each type of milk chain sample. Table 4 shows how 143 samples were collected, comprising 108 milk samples from three different milk chain nodes (farm, middleman, and milk collection center) and 35 milk products (yogurt, cheese, and buttermilk) from the milk product shops.

**Table 4:** The distribution of milk and milk product samples (N = 143) collected at four different buffalo milk chain nodes in six districts of Bangladesh.

District	Farm bulk milk	Middleman	Milk collection center	Milk product shop			Total number of samples
				Yogurt	Cheese	Buttermilk	
Rajshahi	4	1	8	2	3	–	18
Jamalpur	6	4	3	2	–	–	15
Mymensingh	4	3	2	–	–	–	9
Maulvibazar	4	4	8	–	3	2	21
Bhola	5	5	5	10	–	–	25
Noakhali	11	20	11	12	1	–	55
Total	34	37	37	26	7	2	143

### Questionnaire data collection

A questionnaire was developed and divided into four subsections to determine the risk factors associated with the foodborne bacteria related to the buffalo milk chain nodes. Section A captured data at the farm level and included 45 questions. Sections B and C contained 20 questions and collected information from the middlemen and collection centers. Section D included eight questions aimed at gathering information about the milk products, such as the origin of milk, storage time, and the type of containers used. The questionnaire has been detailed in a previous study in this project^25^ and is given as a supplementary file (S1).

### Collection of samples

One hundred and forty-three milk samples were used in this study, where each bulk milk sample was comprised of milk from all the lactating cows in each buffalo farm (n = 34). Two milk samples were collected aseptically at each milk collection center, one before mixing (middleman) (n = 37) and another one after mixing (n = 37). Milk product samples, such as yogurt, cheese, and buttermilk (n = 35), were also collected from the study areas. Samples from the middlemen, milk collection centers, and milk product shops were taken on the same date but were not linked with the source buffalo farm. From each milk sample (bulk milk from farms, middlemen, and milk collection centers), an aliquot of 10 mL and 30–35 g of milk product were aseptically collected in 15 mL and 50 mL falcon tubes, respectively. The sample collection procedure has been described in an earlier study^25^. Upon arrival at the laboratory, the samples were stored on ice immediately and then at − 20 °C.

### Genomic DNA extraction and purification

The milk and milk product samples were subjected to DNA extraction in the Microbial Genetics and Bioinformatics Laboratory, Department of Microbiology, University of Dhaka, Dhaka 1000, Bangladesh. Genomic DNA (gDNA) extraction was performed using a Maxwell^®^ 16 Cell DNA Purification Kit (Promega, UK) with the Maxwell^®^ 16 Instrument platform (Promega, UK). One mL milk sample was centrifuged at 16,000 rcf for 10 min^75,76^. Then, the 200µL of pellet was used for DNA extraction according to the manufacturer’s instructions. For milk products, a 200 mg sample was used for the DNA extraction, following manufacturer instructions as previously described^16,75^. The DNA samples were eluted in a 200 μL elution buffer (Promega, UK) and were stored at − 20 °C until further processing. DNA concentration and purity were analyzed using a NanoDrop 2000 Spectrophotometer (Thermo Fisher Scientific, Waltham, Massachusetts, USA) at a wavelength of 260 nm and A260/A280, respectively. DNA samples were considered appropriate for downstreaming if the DNA concentration was ≥ 9 ng/ µL and they had a 260/280 absorbance ratio (> 1.6 to ≤ 2). Then, the DNA samples were delivered to the Molecular Pathology Laboratory, Department of Veterinary and Animal Sciences (DIVAS), Università degli Studi di Milano (UNIMI), Italy.

### Real-time polymerase chain reaction

Quantitative real-time PCR was carried out using gene-specific primers targeting the examined microbes (Table 5). These primers were selected due to their specificity as described in previous publications^77,78^. Quantitative PCR followed MIQE guidelines^79^ in a final reaction volume of 15 µL using the CFX 96 System (Bio-Rad Laboratories, USA). Each reaction volume contained 7.5µL of 2× Mix EVA Green (SsoFast EvaGreen® Supermix, Bio-Rad Laboratories, USA) and primers specific to the target genes. The PCR reaction was carried out using the same thermal profile for all targets (2 min at 50 °C, 3 min at 95 °C, and 39 cycles of 10 s at 95 °C and 30 s at 60 °C). To assess melting curves, PCR products were incubated at 55 °C for 60 s, and then the temperature was increased to 95 °C at 0.5 °C increments every 10 s. The PCR efficiencies were determined using four-fold serial dilutions of DNA prepared from ATCC strains of the bacteria of interest, such as *S. aureus* ATCC 19048, *E. coli* ATCC 11229, *E. coli* O157:H7 ATCC 35150 (positive for *eae* (Intimin); *stx1* (Shiga toxin 1) positive; *stx2* (Shiga toxin 2) positive), *L. monocytogenes* ATCC 13932, *Y. enterocolitica* DSM 4780, *S. enterica* ATCC 13076, and *C. jejuni* ATCC 33291. ATCC and DSM bacterial strains were obtained from the American-type culture collection (MD, USA) and the German collection of microorganisms and cell cultures (Braunschweig, Germany). No template controls were included in the assays. The results were analyzed using Bio-Rad CFX Maestro 1.0 software (Bio-Rad Laboratories, USA), and the samples with a threshold of CT < 35 cycles were considered positive for the presence of the targeted genes.

**Table 5:** Primer sequences used for RT-PCR to identify the nine target genes for seven foodborne bacteria from the extracted DNA samples.

Bacteria	Gene	Sequences (5ʹ–3ʹ)	Accession number	Amplicon size	Primer concentration^a^	R^2a^	Efficiency (%)^a^
*Staphylococcus aureus*	*htrA*	GAAGTAATATCAGACA AATCAAATCAGTACC	NC_009782	92 bp	500 nM	0.996	86.2
		TCTTCCGGTAAAGTTAATGGCTTCTG					
*Escherichia coli*	*yccT*	GCAGCGTGGTGGCAAAA	CP10315	56 bp	400 nM	1.00	91.6
		CGTGACCACCTTGATTGCAT					
*STEC Escherichia coli*	*eae*	GTAACAATGTCAGAGG CGAGTTG	AE005174	72 bp	600 nM	0.998	92.1
		CCACCGCTTGCTTTCAGTTTAA					
	*stx1*	GGATTTCGTACAACACTGGATGATC	M16625	67 bp	400 nM	0.998	89.9
		GATCAACATCTTCAGCA GTCATTACA					
	*stx2*	ACCCCACCGGGCAGTT	X07865	59 bp	500 nM	0.999	98.3
		CGCGCCTGATAGACATCAAG					
*Listeria monocytogenes*	*inlA*	TAACAGACACGGTCTCG CAAA	CP013288	66 bp	400 nM	0.999	93.9
		TCCCTAATCTATCCGCCTGAAG					
*Yersinia enterocollitica*	*yst*	TGGAGCATTCGGCCAA GAA	X65999	60 bp	400 nM	0.995	92.2
		ATTGGTGTCGATAATG CATCACTGA					
*Salmonella enterica*	*invA*	TGGAAAGGGAAAGCC AGCTT	M90846	68 bp	500 nM	0.995	87.8
		AATAGCGTCACCTTTG ATAAACTTCA					
*Campylobacter jejuni*	*cadF*	TGAACCAAGAGAAGG TGCTTTGT	FJ946061	76 bp	400 nM	0.998	97.5
		AAAACCAAAATGACCTTCCAAAGAAATAGTT					

^a^Primers were selected from previously published studies^77,78^. The appropriate primer concentration was selected based on the reproducibility (R^2^ > 0.99) and efficiency (> 85%) of the selected primers (forward and reverse) to detect the targeted genes specific to each of the ATCC positive controls (*S. aureus* ATCC 19048, *E. coli* ATCC 11229, *E. coli* O157:H7 ATCC 35150, *Listeria monocytogenes* ATCC 13932, *Yersinia enterocolitica* DSM 4780, *Salmonella enterica* ATCC 13076, and *Campylobacter jejuni* ATCC 33291). To calculate R^2^ and efficiency (%), the chosen primers for the targeted genes specific to each ATCC strain were run with duplicates in fourfold serial dilutions (undiluted, 1:3, 1:9, 1:27, 1:81); a negative control was also run (Nuclease free water).

### Statistical analysis

Data analysis was performed using R (version 4.3.1; R Foundation for Statistical Computing, Vienna, Austria). The presence of the targeted genes (*htrA*/ *yccT*/ *eae*/ *stx1*/ *stx2*/ *inlA*/ *yst*/ *invA*/ *cadF*) was a binary outcome variable (yes or no). The prevalence of STEC *E. coli* was calculated based on whether at least one of the entero- and shiga-toxin genes (*eae/ stx1/ stx2*) was present. The prevalence of each pathogen was calculated for each of the milk chain nodes, namely bulk milk, middlemen, milk collection centers, and milk products, by dividing the total number of positives by the total number of samples tested. A chi-squared test was performed to analyse the difference in the prevalence of each pathogen between the four different milk chain nodes. For categorical questionnaire variables, a Fisher’s exact test was performed to compare the difference between the categories of a variable for each RT-PCR positive pathogen. Variables with a P ≤ 0.1 were presented with a prevalence of RT-PCR positive foodborne bacteria and corresponding P-values.

### Supplementary Information

Supplementary Information.

## Data Availability

The dataset used or analyzed in this study will be made available through github link https://github.com/shuvosingha/water-buffalo-foodborne-pathogens-in-Bangladesh upon publication.
