# Supplementary material for: Foodborne bacteria in milk and milk products along the water buffalo milk chain in Bangladesh
Source: Sci Rep. 2024 Jul 19;14:16708. doi: 10.1038/s41598-024-67705-3 (PMC11271598; doi:10.1038/s41598-024-67705-3)
Supplement: Supplementary file 1 — Supplementary Information. [file 41598_2024_67705_MOESM1_ESM.pdf]

Foodborne pathogens in milk and milk products along the water buffalo milk chain in Bangladesh

Shuvo Singha[1,2,3,4], Gerrit Koop[4,5], Md. Mizanur Rahman[3,4], Fabrizio Ceciliani[1,4], Md. Matiar Rahman Howlader[2], Sofia Boqvist[4,6*], Paola Cremonesi[7], M. Nazmul Hoque[4,8], Ylva Persson[4,9], Cristina Lecchi[1,4]

[1]Department of Veterinary Medicine and Animal Sciences, Università degli Studi di Milano, Lodi, 26900, Italy

[2]Department of Physiology, Veterinary, Animal and Biomedical Sciences, Sylhet Agricultural University, Sylhet, 3100, Bangladesh

[3]Department of Medicine and Surgery, Chattogram Veterinary and Animal Sciences University, Chattogram, 4225, Bangladesh

[4]Udder Health Bangladesh, Chattogram, 4225, Bangladesh, www.uhb.org.bd

[5]Sustainable Ruminant Health, Department of Population Health Sciences, Faculty of Veterinary Medicine, Utrecht University, Utrecht, 3584 CL, The Netherlands

[6]Department of Biomedical Sciences and Veterinary Public Health, The Swedish University of Agricultural Sciences, 750 07 Uppsala, Sweden

[7]Institute of Agricultural Biology and Biotechnology, National Research Council, Lodi, 26900, Italy

[8]Department of Gynecology, Obstetrics and Reproductive Health, Bangabandhu Sheikh Mujibur Rahman Agricultural University, Gazipur, 1706, Bangladesh

[9]National Veterinary Institute, Uppsala, 751 89, Sweden

*Correspondence: Sofia Boqvist (Sofia.Boqvist@slu.se)

Survey questionnaire for assessing risk factors associated with the bacteria contamination level of buffalo milk chain, Bangladesh.

Objectives:

1) Identification of different practices at various nodes of the buffalo milk value chain in Bangladesh
2) Assessing the level of contamination of milk/milk products associated with various practices along the buffalo milk value chain in Bangladesh

Sample ID:	Date

A) Study unit: Household/Semibathan/ Bathan/ Intensive/ Semi-Intensive

1. General information

1.1. Name of the Interviewee:			1.2 Mobile no:	
1.3. Gender and Age	☐ Male	☐ Female	☐ Age:	
1.4. Location	Village:	Union:	Upazilla:	
1.5. GPS coordinates	Latitude (Degree):	Longitude (Degree):	Altitude (meters):	
1.6. Title of Interviewee:	☐ Owner	☐ Manager	☐ Milk collector	☐ Other ………...
1.7. Educational status:	☐ Illiterate	☐ Primary	☐ Secondary	☐ Graduation
1.8: Composition of household/bathan/ semibathan	Total no of milking buffalo:	No of heifers:	No of calves:	
	No of cows:	No of bulls:	Average milk yield per day:	
1.9. Selling price of milk				
1.10. Where do you sell your milk?	On-spot sell from farm/ middleman/milk collection center			
1.11. How do you sell your milk?	☐ By own self	☐ Contract basis to middleman	☐ Contract basis to shop	

2. Source and type of sample

2.1. Source of milk	☐ Household	☐ Bathan	☐ Semi-bathan	☐ Semi-intensive	☐ Intensive
2.2 Nodes of value chain	☐ Farm/household /Semi-bathan/Bathan/Semi-intensive/Intensive<br>☐ Trader shipment (at entry)<br>☐ Selling point (Couple of hrs later)				
2.3. Bulk milk somatic cell count (BMSCC)					
2.4. Type of Sample		☐ Milk	☐ Milk Product: curd/ghee/Sweet/milk drink/…………………………		
2.5 Time of sample collection					

3. Milk Container

3.1. Types of container use	☐ Aluminum ☐ Plastic ☐ Others…
3.2.Cleaning milk container with	☐ Hot water ☐ Tubewell water ☐ Tubewell water with detergent ☐ Pond water ☐ Others
3.3. Frequency of cleaning of milk container (per day)?	☐ Once ☐ Twice ☐ Thrice
3.4. Cleanliness score of the milk container	☐ Excellent (no greasiness and dirt inside and outside the container)<br>☐ Good (No greasiness and dirt inside the container)<br>☐ Poor (greasiness and dirt present)
3.5. Do you use a brush or something like that during cleaning the container	☐ Yes ☐ No
3.6. Do you dry the container after cleaning	☐ Yes ☐ No
3.7. If yes, how do you dry the container?	☐ Sun/air dry ☐ Using cloth ☐ Using tissue paper
3.8. Do you keep the bulk milk container open during each milking?	☐ Yes ☐ No
39. If no, what do you use for covering?	
3.10. Do you use anything for sieving milk after milking into the BM container?	☐ Yes ☐ No
3.11 If yes, what do you use?	☐ Cloth ☐ Plastic sieve ☐ Other…

4. Milker's and Buffalo hygiene

4.1. Who does milk buffalo cows?	☐ Owner ☐ Worker
4.2. Do you wash buffalo before milking?	☐ Yes ☐ No
4.3. If yes, which type of water do you use for bathing?	☐ River ☐ Pond ☐ Tube well
4.4. Does the milker wash udder before milking?	☐ Yes ☐ No

4.5. Does the milker dry the udder before milking?	☐ Yes ☐ No
4.6. If yes, how they dry the udder?	☐ Wait for air dry ☐ Using individual cloth<br>☐ Using common cloth ☐ Others…………
4.7Score of milker's hygiene?	☐ 1=Excellent=Milkers use antiseptic and wash hand<br>☐ 2=Good=Milkers only wash hand<br>☐ 3=Poor=Milkers don't wash
4.8. Score of udder hygiene	☐ 1=Excellent=Udder is clean and dried<br>☐ 2=Good=Udder is clean but not dry<br>☐ 3=Poor=Udder is not clean enough
4.9. Do you let the udder be dried before milking?	☐ Yes ☐ No
4.10 If yes, what do you use to dry the udder?	☐ Cloth ☐ Towel ☐ Tissue ☐ Other
4.11. Does any buffalo affect with clinical mastitis (either change in milk, udder, systematic weakness)?	☐ Yes ☐ No
4.12. Do you mix the mastitis milk with normal milk?	☐ Yes ☐ No

5. Storage

5.1. Milk storage at home	☐ Open ☐ Closed
5.2. Storage time before shifting from home?	☐……. day ☐……Min ☐……. Hour
5.3. How do you store milk at home?	☐ Room temp ☐ Cold storage ☐ Freezer
5.4. Duration of milk kept at home	☐ …….min ☐ ……. Hour

6. Some generic question to farmers

9.1 Do you face any problem during milking of your animal?	☐ Yes ☐ No
9.2. Do you face any difficulty to store milk at home before shifting?	☐Yes ☐ No
9.3 Do you have proper transportation facility to shift the milk at shop?	☐Yes ☐ No
9.4. Do you get the proper price by selling milk?	☐Yes ☐No

B) Milk collection point(Trader shipment-at entry)

7. Milk transportation

6.1. Transport milk sample through	
6.2. Types of container use	☐ Aluminum ☐ Plastic ☐ Others…
6.3. Covering material during transport	☐ Cloth ☐ Plastic plate ☐ Aluminum plate ☐ Banana leaves
6.4. Travel time to collection point	☐…….min ☐……hour
6.5. Do you use any material inside the container to prevent milk spoilage during transportation?	☐Yes ☐No
6.6. If yes, what you use?	☐leaves ☐ Ice cubes ☐ Others…
6.7 Trading experience of milk transporter	☐Yes ☐No
6.8 Nature of milk composition?	☐ Mixed milk (cow and buffalo) ☐ Buffalo milk

C) Selling point (After mixing-couple of hours)

7.1 Nature of milk composition?	☐ Mixed milk (cow and buffalo) ☐ Buffalo milk
7.2. Types of container use	☐ Aluminum ☐ Plastic ☐ Others…
7.3. Milk storage at shop	☐Open ☐ Closed
7.4. Milk kept in cold storage/freeze	☐Yes ☐No
7.5. Duration of milk kept at shop	☐…….min ☐……. Hour

D) Milk product

8.1. Types of the product	☐ Curd ☐ Ghee ☐ Sweet ☐ Other
8.2. Where it is made?	☐ At shop ☐ At households ☐ Other
8.3. Storage time	☐……. day ☐……Min ☐……. Hour
8.4. Containers used	☐ Earthen pot ☐ Plastic container ☐ Other
8.5 Type of seller?	☐ Retail seller ☐ Whole seller
8.6 Source of milk purchase	☐ Own shop ☐ Buy milk from others
8.7 Nature of milk composition?	☐ Mixed milk (cow and buffalo) ☐ Buffalo milk
8.8 Milk used	☐ Boiled ☐ Unboiled

Declaration: I have answered all the questions in the interview sheet, and I have full consent about the information given. Best of my Knowledge, the information given by me is correct and can be used in research. If necessary, the researcher can contact me for further information or vice versa in future.

======================   ======================

Interviewer signature   Interviewee signature
